# Fast cross-linking by DOPA2 promotes the capturing of a stereospecific protein complex over nonspecific encounter complexes

**DOI:** 10.52601/bpr.2022.220014

**Published:** 2022-12-31

**Authors:** Jian-Hua Wang, Zhou Gong, Xu Dong, Shu-Qun Liu, Yu-Liang Tang, Xiaoguang Lei, Chun Tang, Meng-Qiu Dong

**Affiliations:** 1 National Institute of Biological Sciences (NIBS), Beijing 102206, China; 2 Tsinghua Institute of Multidisciplinary Biomedical Research, Tsinghua University, Beijing 102206, China; 3 State Key Laboratory of Magnetic Resonance and Atomic Molecular Physics, Innovation Academy for Precision Measurement Science and Technology, Chinese Academy of Sciences, Wuhan 430071, China; 4 State Key Laboratory for Conservation and Utilization of Bio-Resources in Yunnan, Yunnan University, Kunming 650091, China; 5 Beijing National Laboratory for Molecular Sciences, College of Chemistry and Molecular Engineering, Center for Quantitative Biology, Peking-Tsinghua Center for Life Science, Peking University, Beijing 100871, China

**Keywords:** Chemical cross-linking, Mass spectrometry, Transient protein–protein interaction, Encounter complexes, Stereospecific complex, Cross-linker, DOPA2, DSS

## Abstract

Transient and weak protein–protein interactions are essential to many biochemical reactions, yet are technically challenging to study. Chemical cross-linking of proteins coupled with mass spectrometry analysis (CXMS) provides a powerful tool in the analysis of such interactions. Central to this technology are chemical cross-linkers. Here, using two transient heterodimeric complexes EIN/HPr and EIIA^Glc^/EIIB^Glc^ as our model systems, we evaluated the effects of two amine-specific homo-bifunctional cross-linkers with different reactivities. We showed previously that DOPA2 (di-*ortho*-phthalaldehyde with a di-ethylene glycol spacer arm) cross-links proteins 60–120 times faster than DSS (disuccinimidyl suberate). We found that though most of the intermolecular cross-links of either cross-linker are consistent with the encounter complexes (ECs), an ensemble of short-lived binding intermediates, more DOPA2 intermolecular cross-links could be assigned to the stereospecific complex (SC), the final lowest-energy conformational state for the two interacting proteins. Our finding suggests that faster cross-linking captures the SC more effectively and cross-linkers of different reactivities potentially probe protein–protein interaction dynamics across multiple timescales.

## INTRODUCTION

Protein–protein interactions (PPIs) are essential for the proper functions of proteins (La* et al.* 2013). The strength of a PPI is denoted by the equilibrium dissociation constant (*K*_D_), which is equal to *k*_off_/*k*_on_, with *k*_off_ and *k*_on_ being the kinetic dissociation/association rate constants, respectively (Du* et al.* 2016). The window of biologically relevant *K*_D_ values is extremely wide, spanning over 12 orders of magnitude. The PPIs are often classified as transient or stable based on their binding affinities (Perkins* et al.* 2010). Characterizing transient PPIs has been technically challenging (Perkins* et al.* 2010; Qin and Gronenborn 2014). Among the existing techniques, NMR is an exquisite tool for the examination of transient PPIs under physiological conditions (Liu* et al.* 2016; Vaynberg and Qin 2006; Xing* et al.* 2014), but an effective analysis can be hampered by the complexity of NMR spectra that increases with protein size. Other methods such as yeast two-hybrid analysis (Berggard* et al.* 2007), phage display (Smith 1985), affinity-based pull-down (Berggard* et al.* 2007), and fluorescence titration (Acuner Ozbabacan* et al.* 2011) are more successful when applied to the stable interactions than to the transient ones, although they do not provide direct structural information about the binding interfaces.

Chemical cross-linking of proteins coupled with mass spectrometry analysis (abbreviated as CXMS, XL-MS, or CLMS) has become a valuable tool for investigating the structures of protein complexes and PPIs in recent years (Chavez and Bruce 2019; Liu and Heck 2015; O'Reilly and Rappsilber 2018; Wheat* et al.* 2021; Yang* et al.* 2012; Yu and Huang 2018). In general, two residues spatially close to each other can be covalently linked by chemical cross-linkers under mild conditions. Following protease digestion, cross-linked peptide pairs can be identified by tandem mass spectrometry, which then enables the positioning of cross-linked residues in the protein sequences (Herzog* et al.* 2012; Lv* et al.* 2020; Wu* et al.* 2017; Zhao* et al.* 2018, 2019). Both the intra- and inter-protein interaction regions can be further identified by imposing a distance restraint between each pair of cross-linked residues that is no more than the maximal allowable distance of a cross-linker (Tang and Gong 2020). Additionally, CXMS has the advantage of being fast and sensitive and requiring no sample purification to homogeneity (Fan* et al.* 2014; Yu and Huang 2018).

Two interacting proteins can form an ensemble of on-pathway or off-pathway encounter complexes (ECs), which undergo rapid exchange with the stereospecific complex (SC). The SC is mediated by characteristic short-range specific interactions, typically involving hydrogen bonding, salt bridges, hydrophobic stacking, and π-stacking interactions (Fawzi* et al.* 2010; Kozakov* et al.* 2014). In comparison, ECs are formed mainly through nonspecific electrostatic interactions, and are short-lived, lowly populated, and highly heterogeneous (Anthis and Clore 2015; Schilder and Ubbink 2013; Tang* et al.* 2006). Yet by forming the ECs, two binding partners are kept close to each other, facilitating the formation of the SC (Dong* et al.* 2022; Tang* et al.* 2006). Conventional chemical cross-linkers can capture transient PPIs, including both ECs and SC. For example, studies have succeeded in using disuccinimidyl suberate (DSS) and glutaraldehyde to fix transiently formed complexes before affinity purification (Shi* et al.* 2015) or single particle cryo-electron microscopy (Kastner* et al.* 2008).

Cross-linking products are also determined by the physiochemical properties of a chemical cross-linker. These properties include the specificity of the reactive groups of a cross-linker (Belsom and Rappsilber 2021), and length, rigidity, and hydrophobicity/hydrophilicity of the spacer arm (Ding* et al.* 2016; Hofmann* et al.* 2015; Yu* et al.* 2020). Also important is the preferred conformation(s) of a cross-linker, which is related to the chemical composition of the spacer arm and local dielectric constant at the nearby protein surface (Gong* et al.* 2020). Furthermore, it has been tentatively suggested that the reaction kinetics of the reactive group of a cross-linker governs the capturing of a transient versus stable PPI (Belsom and Rappsilber 2021; Yang* et al.* 2018; Ziemianowicz* et al.* 2019), but this remains to be investigated in a formal way.

**Figure 1:**
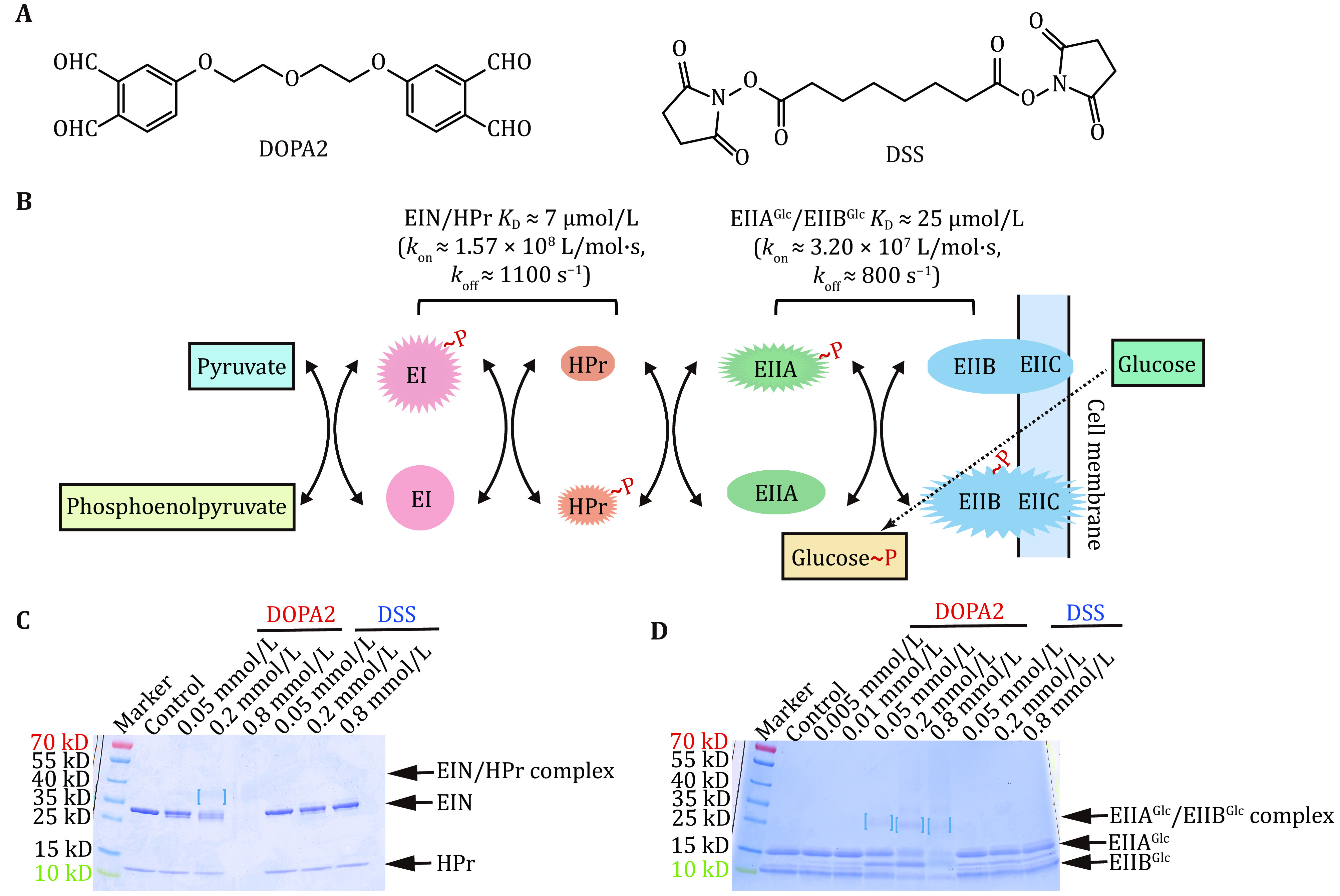
Performance of DOPA2 and DSS on weak protein complexes. **A** The chemical structures of DOPA2 and DSS. **B** Conceptual diagram of carbohydrate transport and phosphorylation by the phosphotransferase system. **C**,**D** SDS-PAGE of DOPA2 or DSS-cross-linked EIN/HPr, EIIA^Glc^/EIIB^Glc^ complexes, with the protein concentrations comparable to the respective equilibrium dissociation constant. Heterodimer bands with the expected molecular weights were marked by square brackets. Cross-linking of EIN/HPr with 0.8 mmol/L DOPA2 resulted in high Mw products that did not enter the separating gel

We recently reported a class of non-hydrolyzable amine-selective di-*ortho*-phthalaldehyde (DOPA) cross-linkers, one of which is DOPA2 (Wang* et al.* 2022). The maximally allowed Cα–Cα distance of DOPA2 is 30.2 Å, slightly longer than that of the widely used N-hydroxysuccinimide (NHS) ester cross-linker DSS (24.0 Å) (Fig. 1A). Importantly, cross-linking of proteins by DOPA2 is 60–120 times faster than that by DSS. We then asked whether DOPA2 outperforms DSS in capturing transient PPIs. In the current study, we compared the cross-linking effects of these two cross-linkers on two heterodimeric complexes. Unexpectedly, we found that whether or not DOPA2 outperforms DSS depends on the nature of a protein complex. The SC was better depicted by the DOPA2 cross-links whereas the more heterogenous and short-lived ECs were better depicted by the DSS cross-links.

## RESULTS

### DOPA2-linked protein complexes were more visible on SDS-PAGE than the DSS-linked counterparts

In the bacterial glucose phosphotransferase system (Deutscher* et al.* 2006; Kotrba* et al.* 2001), the phosphate group from phosphoenolpyruvate is transferred to glucose via Enzyme I (EI), the phosphocarrier protein (HPr), and subsequently, Enzyme II (EII). EI is composed of an N-terminal domain (EIN) and a C-terminal domain (EIC). EII comprises three functional subunits EIIA^Glc^, EIIB^Glc^, and EIIC^Glc^ (Deutscher* et al.* 2006; Kotrba* et al.* 2001). Transient interactions are responsible for the transferring of a phosphoryl group from EIN to HPr (Garrett* et al.* 1997, 1999) and from EIIA^Glc^ to EIIB^Glc^ (Cai* et al.* 2003). The *K*_D_ values of these two protein complexes, EIN/HPr and EIIA^Glc^/EIIB^Glc^, are ~7 μmol/L (*k*_on_ ≈ 1.57 × 10^8^ L/mol·s, *k*_off_ ≈ 1100 s^−1^) (Garrett* et al.* 1997; Suh* et al.* 2007) and ~25 μmol/L (*k*_on_ ≈ 3.20 × 10^7^ L/mol·s,* k*_off_ ≈ 800 s^−1^) (Cai* et al.* 2003; Reizer* et al.* 1992), respectively (Fig. 1B).

Consistent with the transient and weak interactions between EIN and HPr and between EIIA^Glc^ and EIIB^Glc^, DSS cross-linking yielded no discernable covalent dimer bands on the SDS-PAGE at a protein concentration of 0.25 mg/mL (for EIN/HPr) or 0.63 mg/mL (for EIIA^Glc^/EIIB^Glc^) (Fig. 1C and 1D). Note that the protein concentrations used here are in the range for a typical cross-linking experiment. Interestingly, DOPA2 cross-linking at the same protein concentrations generated a faint covalent dimer band for either complex with the expected molecular weight (Fig. 1C and 1D).

**Figure 2:**
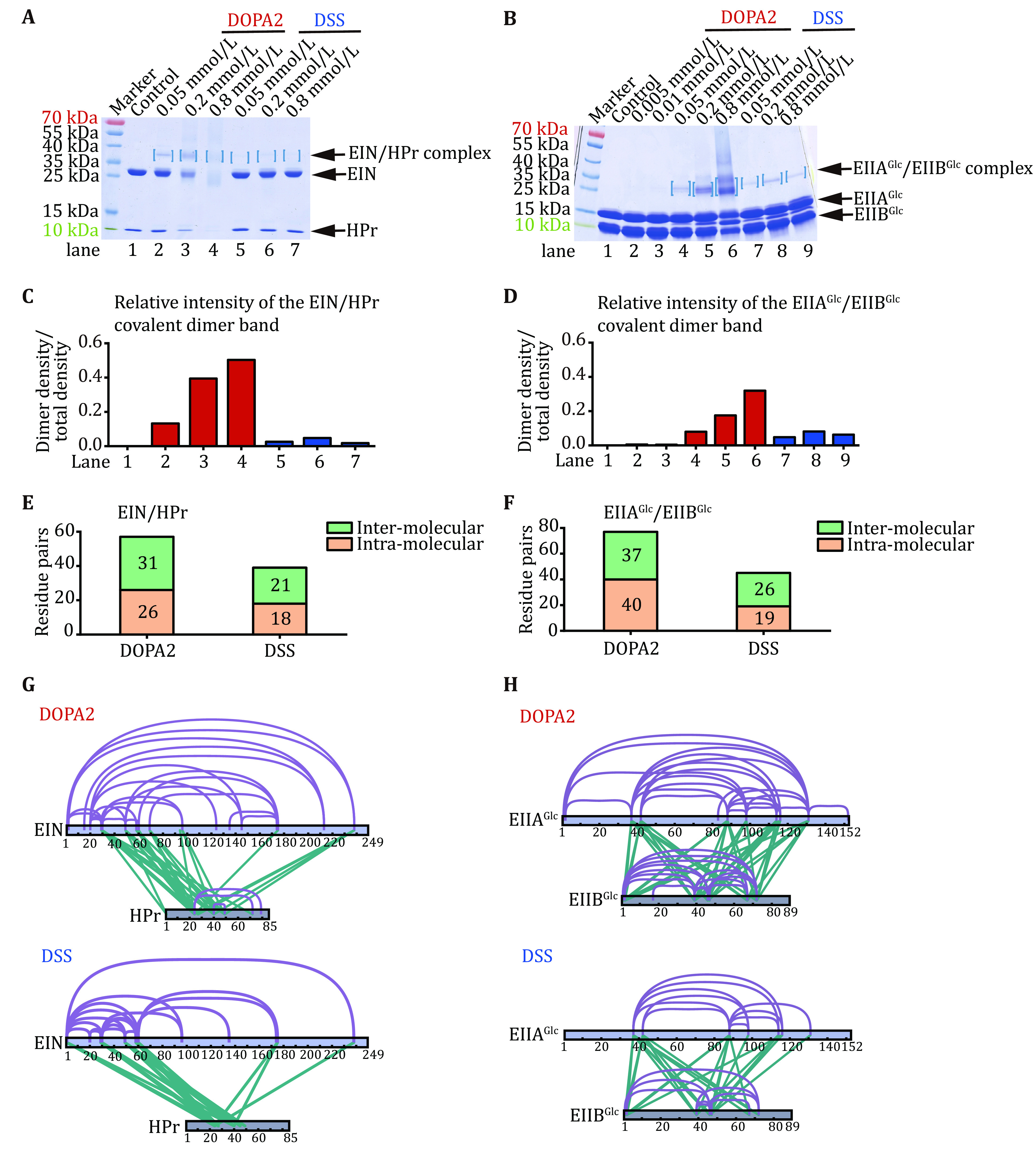
In comparison to DSS, DOPA2 captured more non-covalent dimers readily visible on SDS-PAGE. **A**,**B** SDS-PAGE of cross-linked EIN/HPr and EIIA^Glc^/EIIB^Glc^ complexes. Captured protein heterodimers were marked by square brackets. **C**,**D** The relative intensity of the heterodimer band, normalized by the total intensity. **E**,**F** The inter-molecular or intra-molecular residue pairs identified in the covalent dimer bands cross-linked by DOPA2 or DSS. **G**,**H** DOPA2 cross-linked or DSS cross-linked residue pairs identified from excised dimer bands were mapped to the primary sequences of the respective complexes (visualized using xiNET (Combe* et al.* 2015)). Cross-links were filtered by requiring FDR < 0.01 at the spectra level, E-value < 1 × 10^−3^ and spectral counts ≥ 3

In the above experiments, the protein concentrations were set to about 1 × *K*_D_, *i*.*e*., 7 µmol/L for EIN and HPr (0.06 and 0.19 mg/mL, respectively), and 25 µmol/L for EIIA^Glc^ and EIIB^Glc^ (0.40 and 0.23 mg/mL, respectively). Under these conditions, the equilibrium concentrations of the heterodimers, EIN/HPr and EIIA^Glc^/EIIB^Glc^, were 2.67 and 9.55 μmol/L, respectively. In other words, only 38% of the protein subunits were in the complex form. When the protein concentrations were increased to 10 × *K*_D_, the complex percentage increased to 73%, corresponding to an equilibrium concentration of 51.09 μmol/L for the EIN/HPr heterodimer and 182.46 μmol/L for EIIA^Glc^/EIIB^Glc^. Thus, there is a 19-fold increase in the quantity of the protein complexes relative to that at 1 × *K*_D_. Indeed, at 10 × *K*_D_ concentration, cross-linking reactions with DOPA2 generated more prominent dimer bands on SDS-PAGE than with DSS (Fig. 2A–2D). Therefore, it seems that DOPA2 is better than DSS at capturing transient PPIs.

Liquid chromatography coupled with tandem mass spectrometry analysis (LC-MS/MS) of the cross-linked dimers from the gel bands indicated that the DOPA2 samples contained more intra- and inter-molecular cross-linked residue pairs (referred to as cross-links hereafter) than the DSS counterparts (Fig. 2E–2H). For example, the number of inter-molecular cross-links between EIN and HPr was 31 for DOPA2 and 21 for DSS, and that between EIIA^Glc^ and EIIB^Glc^ was 37 for DOPA2 and 26 for DSS (Fig. 2E and 2F). An analysis of the Euclidean distance of each cross-linked residue pair revealed that a higher percentage of the DOPA2 cross-links (63% for EIN/HPr, 64% for EIIA^Glc^/EIIB^Glc^) are compatible with the known complex structures than the DSS cross-links (28% for EIN/HPr, 51% for EIIA^Glc^/EIIB^Glc^) (Table 1). The same observation holds for the solvent accessible surface distance (SASD) (Table 1).

**Table 1:** Structural compatibility rate of residue pairs (inter-links plus intra-links)

	Cα–Cα distance (Å)	EIN/HPr			EIIA^Glc^/EIIB^Glc^	
		Euclidean distance	SASD		Euclidean distance	SASD
DOPA2	30.2*	63% (36/57)	46% (24/52)		64% (35/55)	35% (18/52)
DSS	24	28% (11/39)	18% (6/34)		51% (18/35)	30% (10/33)
* Spacer arm with MM2 minimization SASD is short for Solvent Accessible Surface Distance. Structural models of EIN/HPr (PDB code: 3EZA) and EIIA^Glc^/EIIB^Glc^ (PDB code: 1O2F) were used as reference. Both were treated as stereospecific complexes						

### Transient protein complexes cross-linked by DSS were heterogeneous and spreading widely on SDS-PAGE

**Figure 3:**
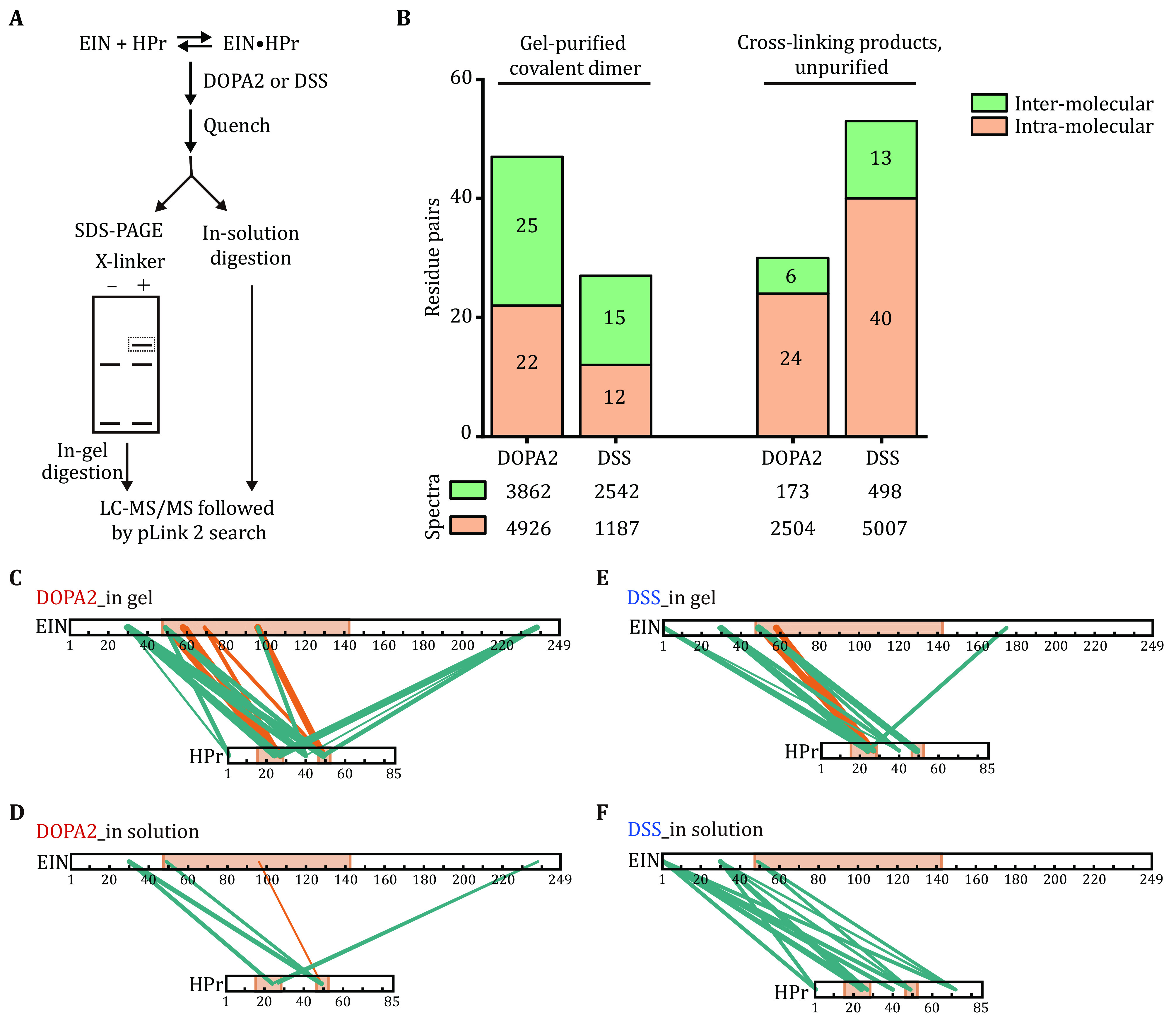
Contrasting cross-link identification results before and after SDS-PAGE purification. **A** Schematic diagram of in-gel digestion or in-solution digestion of EIN/HPr complex after DOPA2 or DSS cross-linking. **B** The inter-molecular or intra-molecular residue pairs identified in the EIN/HPr complex cross-linked by DOPA2 or DSS with purification by SDS-PAGE or not. The number of spectra identified is shown below. **C**,**D** The inter-molecular residue pairs identified by DOPA2 in the dimer band or in solution were mapped to the primary sequence of EIN/HPr (visualized using xiNET (Combe* et al.* 2015)). **E**,**F** As in panels C and D, but for cross-links identified by DSS. The stereospecific interface between EIN and HPr was indicated by light orange color. The green lines and the orange lines denoted the cross-links that were assigned to ECs and SC, respectively. The numbers of the corresponding spectra of each cross-link were indicated by the thickness of the line. Cross-links were filtered by requiring FDR < 0.01 at the spectra level, E-value < 1 × 10^-8^ and spectral counts > 3

In parallel, we analyzed the same cross-linking reactions without SDS-PAGE separation (Fig. 3A). Interestingly, when digested in solution, the samples cross-linked with DSS yielded more cross-link identifications than the samples cross-linked with DOPA2 for both intra- and inter-molecular cross-links (*i*.*e*., 40 and 13 for DSS, 24 and 6 for DOPA2) (Fig. 3B). This is the opposite of the in-gel digestion result, which contained fewer DSS cross-links than DOPA2 cross-links. Furthermore, unlike the samples digested in-solution, the excised, in-gel digested covalent dimers produced more inter- than intra-molecular cross-links, either with DOPA2 or with DSS (Fig. 3B). When the inter-molecular cross-links were mapped to the known structure of EIN/HPr, we found that the cross-links bridging the interface between EIN and HPr are more for DOPA2 than for DSS (Fig. 3C–3F, denoted by orange-colored lines). Of note, the cross-links examined here all passed a stringent filter of the search results (FDR < 0.01, at least four MS2 spectra at E-value < 1 × 10^−8^).

**Figure 4:**
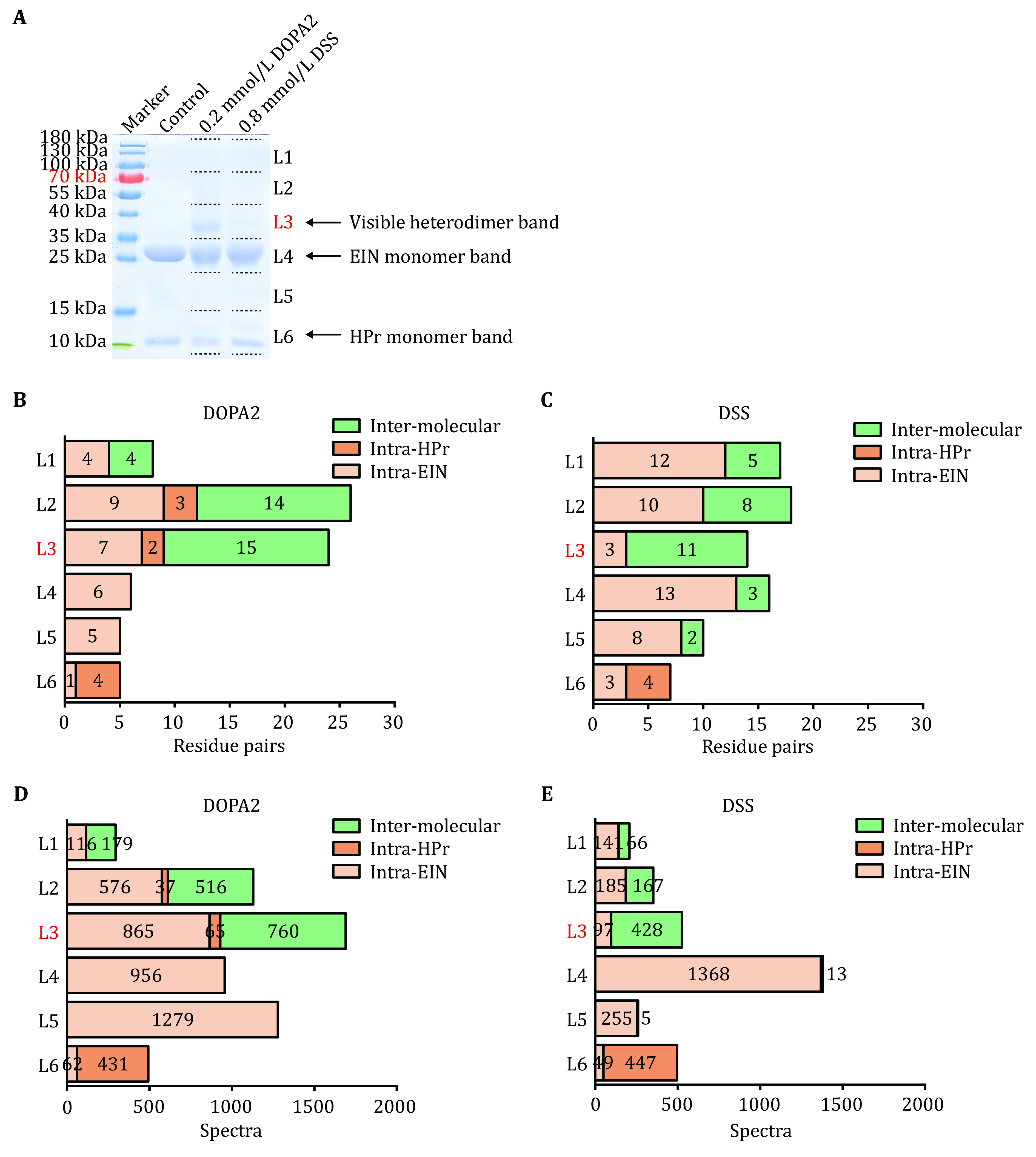
Systematic analysis of cross-linked protein species separated by SDS-PAGE. **A** SDS-PAGE of DOPA2 or DSS-cross-linked EIN/HPr and the demarcation range for systematic excision of the gel slides, arbitrarily denoted as L1 through L6. **B**,**C** The number of inter- or intra-molecular residue pairs identified for DOPA2 or DSS cross-linking in L1–L6, respectively. EIN(30)-EIN(30), the only unambiguously identified homodimeric cross-link suggestive of higher-order interactions between the EIN/HPr heterodimers, was included in this graph. It came from L1 and L2 of the DOPA2 treated sample or L1, L2 and L4 of the DSS treated sample. **D**,E The number of inter- or intra-molecular spectra identified for DOPA2 or DSS cross-linking in L1–L6, respectively. Cross-links were filtered by requiring FDR < 0.01 at the spectra level, E-value < 1 × 10^-8^ and spectral counts in each sample ≥ 2

To account for the discrepancy, we systematically analyzed the cross-linking products following SDS-PAGE separation. The most prominent covalent dimer band (L3) and the gel slices above (denoted as L1 and L2) and below (denoted as L4, L5, and L6) were all analyzed (Fig. 4A). For intra-molecular cross-links (intra-EIN and intra-HPr), more cross-links were identified for DSS than for DOPA2 (53 versus 41) from these gel slice samples (Fig. 4B and 4C). For inter-molecular cross-links between EIN and HPr, the opposite was observed: fewer inter-molecular cross-links were identified with DSS than with DOPA2 (29 versus 33 residue pairs, or 679 versus 1455 spectra) (Fig. 4B and 4C, and Fig. 4D and 4E). Notably, the inter-molecular cross-links by DOPA2 were all identified from either the visible EIN/HPr dimer band (L3) or above (L1–L2), whereas those by DSS were detected throughout the lane (L1–L5) (Fig. 4B and 4C). These results suggest that, when compared to the dimeric complexes cross-linked by DOPA2, DSS-linked complexes are more heterogeneous, spreading over a wider molecular weight range and affording a less distinct dimeric band.

### DOPA2 cross-linking favored the stereospecific complex whereas DSS cross-linking favored the encounter complexes

We showed in our previous work (Gong* et al.* 2015) that a vast majority of the EIN/HPr inter-molecular cross-links generated by BS^2^G or BS^3^ (both are NHS ester cross-linkers) featured ECs. Only one out of the 13 inter-molecular BS^2^G or BS^3^ cross-links comes from SC (Gong* et al.* 2015). Fleeting ECs cannot be captured by crystallization, nor by the standard NMR technique, but are detectable with the use of paramagnetic NMR (Tang* et al.* 2006). In contrast, the SC of EIN/HPr has been determined to be atomic resolution using the standard NMR method (Garrett* et al.* 1999; Tang* et al.* 2006).

**Figure 5:**
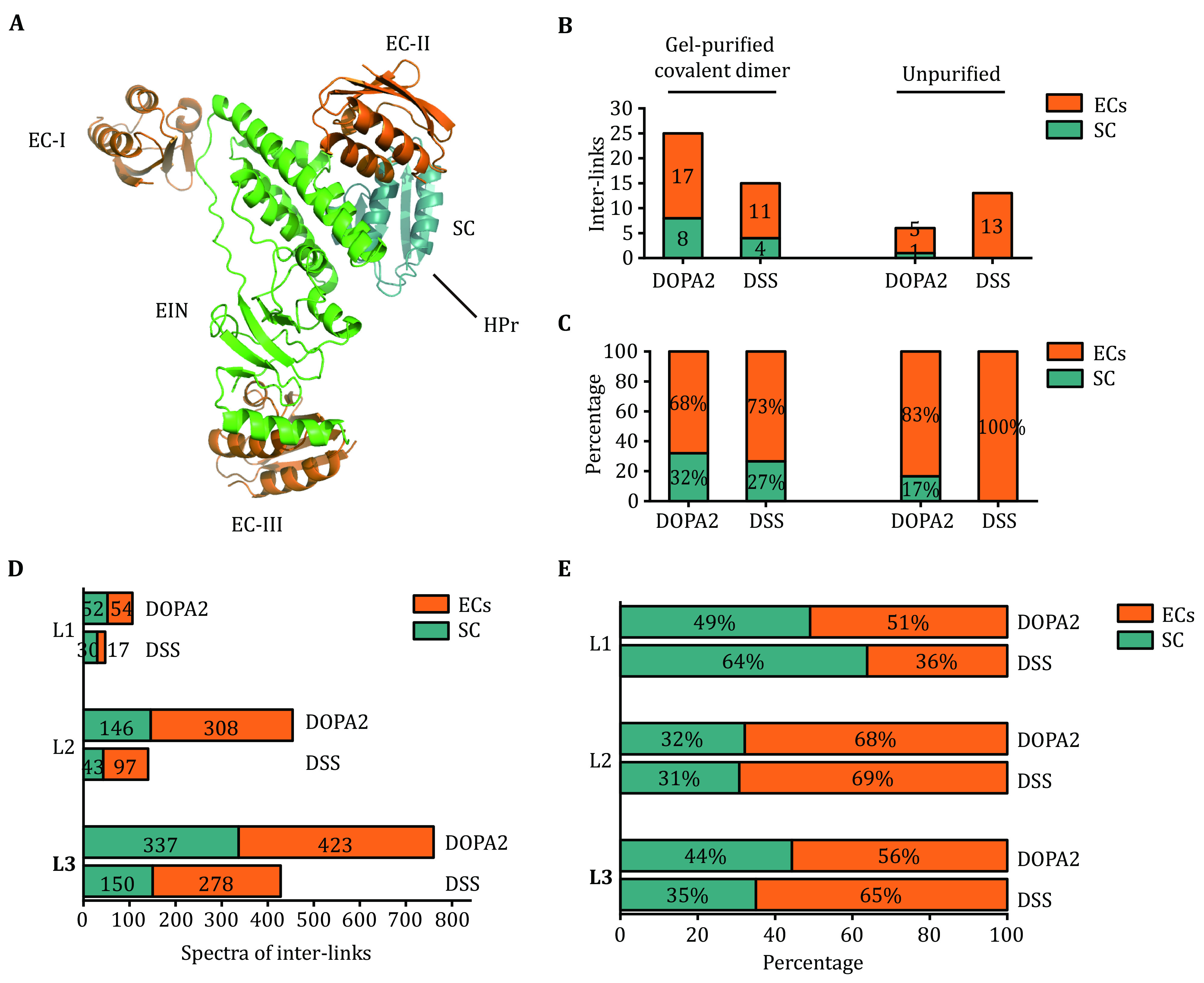
Analysis of the heterodimeric cross-links with respect to the conformations of the stereospecific and encounter complexes. **A** Three representative encounter structures (EC-I, EC-II, and EC-III) and stereospecific complex (SC, PDB code: 3EZA) (Fawzi* et al.* 2010; Garrett* et al.* 1999; Tang* et al.* 2006) for EIN/HPr complex. **B** The number of DOPA2 or DSS cross-linked inter-links of EIN/HPr compatible with SC and ECs *w*/*t* or *w*/*o* gel purification. **C** The relative percentages of DOPA2 or DSS cross-linked inter-links of EIN/HPr compatible with SC and ECs *w*/*t* or *w*/*o* gel purification. **D** The number of DOPA2 or DSS cross-linked spectra of EIN/HPr compatible with SC and ECs in L1–L3, respectively. **E** The relative percentages of DOPA2 or DSS cross-linked spectra of EIN/HPr compatible with SC and ECs in L1–L3, respectively. Cross-links were filtered by requiring FDR < 0.01 at the spectra level, E-value < 1 × 10^−8^ and spectral counts in each sample ≥ 2

In the current study, we mapped inter-molecular cross-links on the representative structures of the ECs and the SC of EIN/HPr (Fig. 5A). When the EIN/HPr samples were analyzed by LC-MS/MS without SDS-PAGE separation (in-solution samples) as done before (Gong* et al.* 2015), one of the six inter-molecular DOPA2 cross-links is consistent with the SC (17%). In contrast, none of the 13 inter-molecular DSS cross-links is consistent with the SC (Fig. 5B, Table 2 and the supplementary Table S1A and S1B). From the gel band of the covalently linked EIN/HPr dimer, four (27%) out of the 15 inter-molecular DSS cross-links represented the SC (Fig. 5C, Table 2 and the supplementary Table S1C). As the SC cross-links are already favored by DOPA2, further increase in SC became less obvious when analyzing the gel band (Fig. 5C and Table 2). Nevertheless, LC-MS/MS analysis of the gel bands containing DOPA2-linked EIN/HPr showed that 8 out of 25 (32%) inter-molecular cross-links correspond to the SC (Fig. 5C, Table 2 and the supplementary Table S1D). In brief, cross-links representing the SC were found enriched among the DOPA2 cross-links and the cross-links identified from the exercised covalent complex bands.

**Table 2:** Inter-molecular cross-links identified from the EIN/HPr complex

	Cross-linker		Encounter complexes (ECs)			Stereospecific complex (SC)		Total X-links	Total spectra of inter-molecular X-links	Total spectra of intra-molecular X-links
			# of X-links	# of spectra		# of X-links	# of spectra			
In-solution	DSS		13 (100%)	498 (100%)		0	0	13	498	5007
	DOPA2		5 (83%)	168 (97%)		1 (17%)	5 (3%)	6	173	2504
In-gel	DSS		11 (73%)	1515 (60%)		4 (27%)	1027(40%)	15	2542	1187
	DOPA2		17 (68%)	2186 (56.6%)		8 (32%)	1676 (43.4%)	25	3862	4926
The cross-links identification results were filtered by requiring FDR < 0.01 at the spectra level, E-value < 1 × 10^−8^ and spectral counts > 3. The estimated FDR at the residue pair level is zero. The cross-links were classified according to their structural compatibility with either the stereospecific complex or the encounter complexes. The cross-linking data of 0.05, 0.2, and 0.8 mmol/L DOPA2 or DSS were combined									

We also examined the inter-molecular cross-links identified in gel slices L1–L3. Starting with the DOPA2- or DSS-linked covalent dimer bands at L3, more DOPA2 cross-links (six residue pairs, 337 spectral counts) were consistent with the SC than DSS cross-links (five residue pairs, 150 spectral counts) (Fig. 5D and 5E and the supplementary Table S2). This is consistent with the result shown above (Fig. 5B and 5C) and with that from L2 (Fig. 5D and 5E, and the supplementary Table S2). The highest molecular weight region L1 had only a small number of cross-link spectra identified, diminishing but not eliminating the said difference between DOPA2 and DSS (Fig. 5D and the supplementary Table S2). Together, these data indicate that DOPA2 captures the SC more effectively than DSS.

### A proposed model for the differential preference of SC and ECs by DOPA2 and DSS cross-linking

One intriguing result in this study is the differential preference of DOPA2 and DSS towards SC and EC, respectively. Namely, for the weak EIN/HPr complex, the products generated by the relatively slow cross-linking reagent DSS predominantly represent the fleeting ECs, while the longer-lived SC is better represented among the products of the faster cross-linking reagent DOPA2. Similar to this observation, a previous study also demonstrated that NHS esters DSS/BS^3^ trapped dynamic states in the ensemble while the fast photoactivatable diazirine-containing SDA cross-linker had better performance than DSS/BS^3^ for accurate modeling (Ziemianowicz* et al.* 2019).

The process of capturing a protein–protein interaction by cross-linking may be conceptualized as follows. We start from the intermediate state when one end of the cross-linker is already covalently attached to either protein (A or B) in a transient complex A•B. For the subsequent inter-molecular cross-linking reaction, the likelihood of the formation of an inter-molecular cross-link will be determined by the reaction rate of the amine-targeting group as well as the half-life of the A•B complex.

On the other hand, if the reaction rate is not fast enough in comparison to the half-life of the A•B complex, no inter-molecular cross-link will form in this round of association. However, this “planted” cross-linker still has a chance to form an inter-molecular cross-link in the next round of association before it loses reactivity following an attack from an adjacent intra-molecular amine group or a quenching reagent.

**Figure 6:**
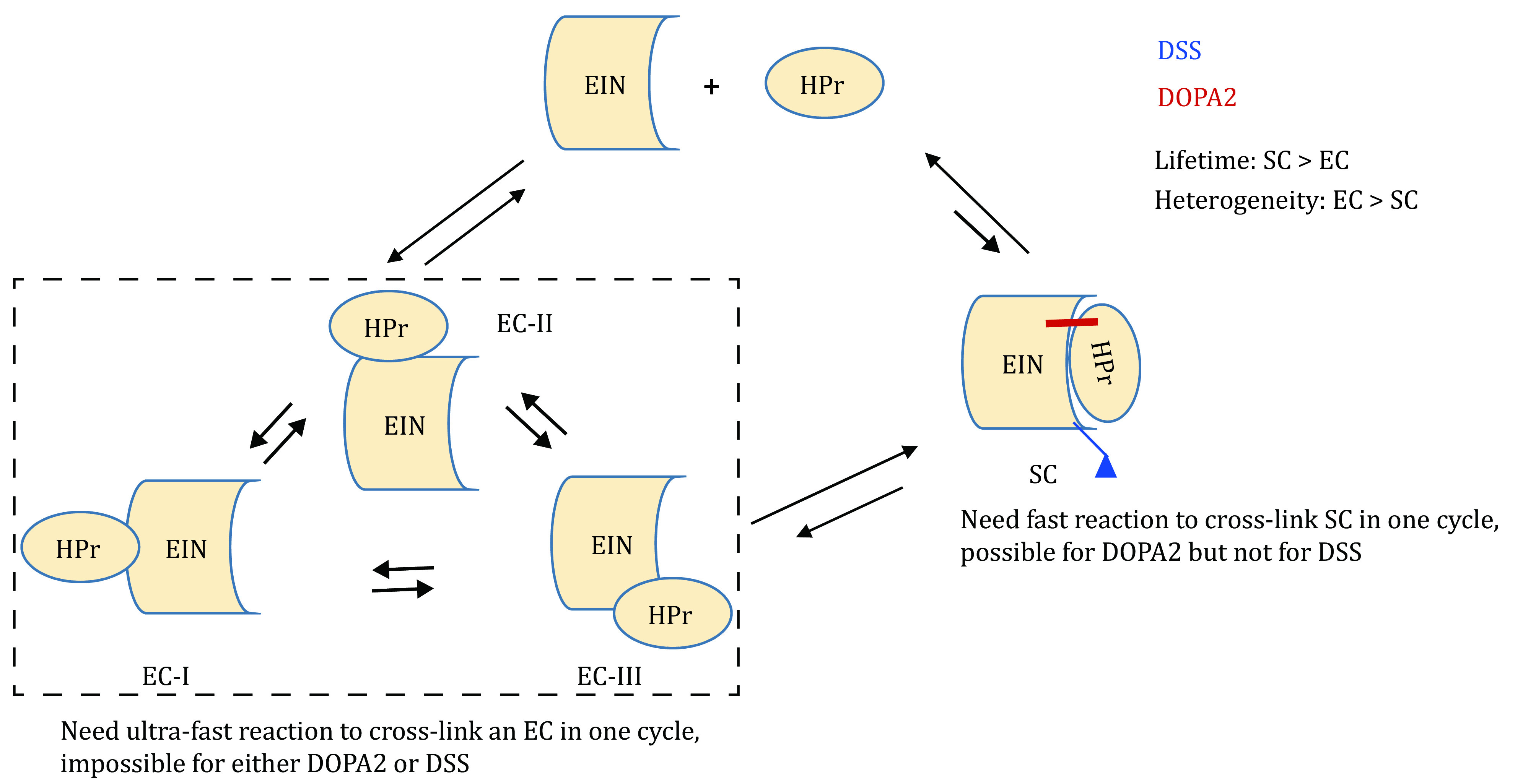
Proposed model for the conformational preference by DOPA2 and DSS in cross-linking. (1) An EC is too transient for either DOPA2 or DSS to capture immediately. (2) Compared to DSS, the fast-reacting cross-linker DOPA2 has a higher chance of capturing the stereospecific complex of EIN/HPr, by forming an inter-molecular cross-link before the two subunits dissociate. (3) Cross-linking by DSS is too slow to capture an EIN/HPr complex in one shot. DSS cross-linking probably takes place in two steps: first a DSS mono-link forms, then in one or more subsequent dissociation/association cycles, the mono-link planted on a subunit turns into a cross-link. Between the mono-link and the cross-link, the protein has many opportunities to sample various alternative conformational states, including the ECs

As shown in Fig. 6, we propose that DSS or BS^3^ cross-linking reactions are too slow to capture either the SC or the fleeting ECs in a single association/dissociation round. As a result, the observed cross-links may come from the subsequent association events. As the SC and the ECs undergo rapid interconversion, it is not surprising that a DSS or BS^3^ mono-linked protein can be attached to its partner protein in conformations different from the SC. This is in line with an earlier work demonstrating that a slow reacting sulfonyl fluoride group can capture weak and transient interactions once planted onto a protein (Yang* et al.* 2018). This explains the observation that the number of inter-molecular DSS or BS^3^ cross-links identified from EIN/HPr is small and most of them represent ECs.

In contrast, DOPA2 has a faster reaction rate and therefore can be more efficient in capturing the SC before the two subunits dissociate. Our recent NMR analysis showed that the actual dissociation constant *k*_off_ of EIN/HPr complex can reach 8900 s^−1^, corresponding to a lifetime of ~100 µs of the complex (Dong* et al.* 2022). Thus, it is remarkable that DOPA2, or DSS to a lesser extent, still captures the stereospecific EIN/HPr complex. As ECs exist as an ensemble of conformations while the SC represents the final lowest-energy conformational state, cross-linked ECs are more heterogeneous, and therefore more spread-out and less visible on SDS-PAGE, as in the case of DSS cross-links.

## DISCUSSION

In this study, we have shown that under near-physiological conditions, DOPA2 is advantageous over DSS in capturing transiently interacting proteins for the structural characterization of a stereospecific complex. It makes intuitive sense that a faster cross-linker is advantageous in capturing a transient PPI, but the truth is more nuanced than expected. “Fast” and “slow” are relative terms. Although faster than DSS in protein cross-linking, DOPA2 is probably not fast enough to cross-link the SC of EIN/HPr whose lifetime is ~100 µs in a single association-dissociation cycle, let alone the fleeting ECs. Counterintuitively, the slower DSS captures more ECs, probably by going through more protein association-dissociation rounds than DOPA2. This could explain the observation that although most intermolecular cross-links by either DOPA2 or DSS were consistent with the ECs, more DOPA2 intermolecular cross-links than DSS intermolecular cross-links could be assigned to the SC (see Table 2). It is worth noting that for a stable protein-protein interaction, slow NHS ester cross-linkers can capture the SC effectively (Gong 2015).

Though the structures of ECs of EIIA^Glc^/EIIB^Glc^ are not yet available from other characterization methods and cannot be compared against, the cross-links found outside the interface region of the SC are in highly charged patches, characteristic of ECs. Further, DSS and DOPA2 displayed the same contrasting behaviors on EIIA^Glc^/EIIB^Glc^ as they did on EIN/HPr. For either heterodimeric complex, more DOPA2 cross-links (>60%, with intra- and inter-molecular cross-links combined) were consistent with the structure of the SC than did the DSS cross-links (28%–51%) (Table 1). Moreover, DOPA2 cross-linking generated a covalent heterodimer band that is much more distinct on SDS-PAGE than did DSS cross-linking for both EIN/HPr and EIIA^Glc^/EIIB^Glc^ (Fig. 1 and Fig. 2). Thus, our findings lead to the conclusion that gel purification is generally a good practice for structural modeling of the SC.

We also noticed that in our previous analysis of six model proteins (aldolase, BSA, catalase, GST, lysozyme, and myosin), the DOPA2 cross-links have a higher compatibility rate with the determined structures of these monomeric or homo-dimeric proteins than the DSS cross-links (Wang* et al.* 2022). Based on our current findings, we propose that the difference in the compatibility rate can be accounted for, at least in part, by the fact that faster DOPA2 cross-linking is more effective at capturing the lowest-energy conformational state, thereby accommodating intramolecular dynamics.

## EXPERIMENTAL SECTION

### Materials and reagents

DSS, tris(2-carboxyethyl) phosphine (TCEP), Dithiothreitol (DTT), and 2-Iodoacetamide (IAA) were purchased from Pierce Biotechnology (Thermo Scientific). Dimethylsulfoxide (DMSO), HEPES, NaCl, urea, CaCl_2,_ and methylamine were purchased from Sigma-Aldrich. Acetonitrile (ACN), formic acid (FA), acetone, and ammonium bicarbonate were purchased from J.T. Baker. Trypsin and Asp-N (gold mass spectrometry grade) were purchased from Promega. DOPA2 was synthesized as previously described (Wang* et al.* 2022).

### Preparation of protein samples

The N-terminal domain of *E. coli* enzyme I (EIN, residues 1-249) and the histidine-containing phosphor carrier protein HPr, EIIA^Glc^ and EIIB^Glc^ were purified as previously described (Garrett* et al.* 1999; Xing* et al.* 2014). Eluted proteins were exchanged into 20 mmol/L HEPES, 150 mmol/L NaCl, pH 7.4.

### Protein cross-linking

The *K*_D_ value of EIN/HPr complex was ~7 μmol/L in 20 mmol/L Tris-HCl buffer, pH 7.4, 150 mmol/L NaCl at 40 ˚C (Suh* et al.* 2007). EIN/HPr complexes were diluted to 1 × *K*_D_ (0.25 mg/mL) and 10 × *K*_D_ (2.5 mg/mL), and then were cross-linked with DOPA2 at the final concentration of 0.05, 0.2 and 0.8 mmol/L at room temperature for 10 min, respectively. EIN/HPr complexes were also cross-linked with DSS at the final concentration of 0.05, 0.2 and 0.8 mmol/L at room temperature for 1 h.

The *K*_D_ value of EIIA^Glc^/EIIB^Glc^ complex was estimated to be ~25 μmol/L. According to a previous study, EIIA^Glc^/EIIB^Glc^ has a *K*m value of 1.7–25 μmol/L (Reizer* et al.* 1992). We did not succeed in obtaining an accurate *K*_D_ value by either surface plasmon resonance assay or ELISA. However, it is clear that the binding is weak, and the *K*_D_ value is likely greater than 10 μmol/L. EIIA^Glc^/EIIB^Glc^ complexes were diluted to 1 × *K*_D_ (0.63 mg/mL) and 10 × *K*_D_ (6.3 mg/mL), and then were cross-linked with DOPA2 at the final concentration of 0.005, 0.01, 0.05, 0.2 and 0.8 mmol/L at room temperature for 10 min, respectively. EIIA^Glc^/EIIB^Glc^ complexes were also cross-linked with DSS at the final concentration of 0.05, 0.2 and 0.8 mmol/L at room temperature for 1 h.

### In-solution digestion

Cross-linked proteins were precipitated with the 4-fold volume of acetone for at least 30 min at −20 °C. The pellets were air dried and then dissolved, assisted by sonication, in 8 mol/L urea, 20 mmol/L methylamine, 100 mmol/L Tris, pH 8.5. After reduction (5 mmol/L TCEP, RT, 20 min) and alkylation (10 mmol/L IAA, RT, 15 min in the dark), the samples were diluted to 2 mol/L urea with 100 mmol/L Tris, pH 8.5. Denatured proteins were digested by trypsin alone or trypsin plus Asp-N at a 1/50 (*w*/*w*) enzyme/substrate ratio at 37 °C for 16–18 h, and the reactions were quenched with 5% formic acid (final conc.).

### In-gel digestion

The target bands in the one-dimensional glycine gel were excised manually from the gel slab and cut into pieces. Briefly, after fully washed with the destaining solution and ddH_2_O, the gel pieces were in-gel reduced (10 mmol/L DTT, 56 °C, 40 min) and alkylated (55 mmol/L IAA, RT, 60 min in the dark) and then dehydrated by 100% ACN. Gel pieces were further rehydrated (50 mmol/L NH_4_HCO_3_, 10 ng/μL trypsin) and digested for 16–18 h. The peptides were twice extracted from the gel by extraction solution I (50% ACN, 5% FA) and extraction solution II (75% ACN, 5% FA), respectively. The extracted digests were combined and the sample volume was reduced to about 10 μL in SpeedVac for MS analysis.

### LC-MS analysis

All proteolytic digestions of proteins were analyzed using an EASY-nLC 1000 system (Thermo Fisher Scientific) interfaced with an HF Q-Exactive mass spectrometer (Thermo Fisher Scientific). Peptides were loaded on a pre-column (75 μm ID, 4 cm long, packed with ODS-AQ 12 nm–10 μm beads) and separated on an analytical column (75 μm ID, 12 cm long, packed with Luna C18 1.9 μm 100 Å resin). Slight modifications to the separation method were made for different samples. EIN/HPr and EIIA^Glc^/EIIB^Glc^ complexes were injected and separated with a 75 min linear gradient at a flow rate of 200 nL/min as follows: 0–5% B in 1 min, 5%–35% B in 59 min, 35%–100% B in 5 min, 100% B for 10 min (A = 0.1% FA, B = 100% ACN, 0.1% FA). The top fifteen most intense precursor ions from each full scan (resolution 60,000) were isolated for HCD MS2 (resolution 15,000; NCE 27) with a dynamic exclusion time of 30 s. Precursors with 1+ , 2+ , more than 6+ , or unassigned charge states were excluded.

### Identification of cross-links with pLink 2

The search parameters used for pLink 2 (Chen* et al.* 2019) were as follows: instrument, HCD; precursor mass tolerance, 20 ppm; fragment mass tolerance 20 ppm; cross-linker DOPA2 (cross-linking sites K and protein N-terminus, cross-link mass-shift 334.084, mono-link *w*/*o* hydrazine mass-shift 352.096, mono-link *w*/*t* hydrazine mass-shift 348.111), cross-linker DSS (cross-linking sites K and protein N-terminus, cross-link mass-shift 138.068, mono-link mass-shift 156.079); fixed modification Carbamidomethyl[C]; variable modifications Deamidated[N], Deamidated[Q], and Oxidation[M]; peptide length, minimum 6 amino acids and maximum 60 amino acids per chain; peptide mass, minimum 600 and maximum 6,000 Da per chain; enzyme, trypsin, with up to three missed cleavage sites per cross-link. Protein sequences of model proteins were used for database searching. The results were filtered by requiring a spectral false identification rate < 0.01.

### Cα–Cα distance calculations

The Cα–Cα Euclidean distances were measured using PyMOL for each PDB file. The PDB files we use are as follows: EIN/HPr (3EZA (Garrett* et al.* 1999)), EIIA^Glc^/EIIB^Glc^ (1O2F (Cai* et al.* 2003)). The Cα–Cα Solvent Accessible Surface Distance (SASD) was calculated using Jwalk (Matthew Allen Bullock* et al.* 2016). If the SASD of cross-linked residue pairs cannot be calculated due to a lack of surface accessibility, these residue pairs are excluded from the calculation. When calculating structural compatibility, the distance cut-offs are 30.2 Å for DOPA2, and 24.0 Å for DSS.

### Classification of inter-molecular cross-links

The ensemble structure refinement based on the intermolecular CXMS restraints of BS^2^G and BS^3^ was performed in our previous study (Gong* et al.* 2015). The ensemble structures of EC-I, EC-II, and EC-III from the previous report (Tang* et al.* 2006) and the SC from the PDB structure (accession code 3ZEA) were used as the reference structures. If the Euclidean distance of cross-linked residue pairs was within the range of the cross-linker, the cross-link was assigned to the SC. The cross-link that would be over-length in the SC was assigned to the ECs.

## Conflict of interest

Jian-Hua Wang, Zhou Gong, Xu Dong, Shu-Qun Liu, Yu-Liang Tang, Xiaoguang Lei, Chun Tang and Meng-Qiu Dong declare that they have no conflict of interest.

## SUPPLEMENTARY DATA

Supplementary data to this article can be found online.

## References

[bAcuner2011] (2011). Transient protein-protein interactions. Protein Eng Des Sel.

[bAnthis2015] (2015). Visualizing transient dark states by NMR spectroscopy. Q Rev Biophys.

[bBelsom2021] (2021). Anatomy of a crosslinker. Curr Opin Chem Biol.

[bBerggard2007] (2007). Methods for the detection and analysis of protein-protein interactions. Proteomics.

[bCai2003] (2003). Solution structure of the phosphoryl transfer complex between the signal-transducing protein IIAGlucose and the cytoplasmic domain of the glucose transporter IICBGlucose of the *Escherichia coli* glucose phosphotransferase system. J Biol Chem.

[bChavez2019] (2019). Chemical cross-linking with mass spectrometry: a tool for systems structural biology. Curr Opin Chem Biol.

[bChen2019] (2019). A high-speed search engine pLink 2 with systematic evaluation for proteome-scale identification of cross-linked peptides. Nat Commun.

[bCombe2015] (2015). xiNET: cross-link network maps with residue resolution. Mol Cell Proteomics.

[bDeutscher2006] (2006). How phosphotransferase system-related protein phosphorylation regulates carbohydrate metabolism in bacteria. Microbiol Mol Biol Rev.

[bDing2016] (2016). Increasing the depth of mass-spectrometry-based structural analysis of protein complexes through the use of multiple cross-Linkers. Anal Chem.

[bDong2022] (2022). Preferential interactions of a crowder protein with the specific binding site of a native protein complex. J Phys Chem Lett.

[bDu2016] (2016). Insights into protein-ligand interactions: mechanisms, models, and methods. Int J Mol Sci.

[bFan2014] (2014). A new approach to protein structure and interaction research: chemical cross-linking in combination with mass spectrometry. Prog Biochem Biophys.

[bFawzi2010] (2010). Mechanistic details of a protein-protein association pathway revealed by paramagnetic relaxation enhancement titration measurements. Proc Natl Acad Sci USA.

[bGarrett1997] (1997). Identification by NMR of the binding surface for the histidine-containing phosphocarrier protein HPr on the N-terminal domain of enzyme I of the Escherichia coli phosphotransferase system. Biochemistry.

[bGarrett1999] (1999). Solution structure of the 40,000 Mr phosphoryl transfer complex between the N-terminal domain of enzyme I and HPr. Nat Struct Biol.

[bGong2015] (2015). Visualizing the ensemble structures of protein complexes using chemical cross-linking coupled with mass spectrometry. Biophys Rep.

[bGong2020] (2020). The conformational preference of chemical cross-linkers determines the cross-linking probability of reactive protein residues. J Phys Chem B.

[bHerzog2012] (2012). Structural probing of a protein phosphatase 2A network by chemical cross-linking and mass spectrometry. Science.

[bHofmann2015] (2015). Protein structure prediction guided by crosslinking restraints-a systematic evaluation of the impact of the crosslinking spacer length. Methods.

[bKastner2008] (2008). GraFix: sample preparation for single-particle electron cryomicroscopy. Nat Methods.

[bKotrba2001] (2001). Bacterial phosphotransferase system (PTS) in carbohydrate uptake and control of carbon metabolism. J Biosci Bioeng.

[bKozakov2014] (2014). Encounter complexes and dimensionality reduction in protein-protein association. Elife.

[bLa2013] (2013). Predicting permanent and transient protein-protein interfaces. Proteins.

[bLiu2015] (2015). Interrogating the architecture of protein assemblies and protein interaction networks by cross-linking mass spectrometry. Curr Opin Struct Biol.

[bLiu2016] (2016). Transient protein-protein interactions visualized by solution NMR. Biochim Biophys Acta.

[bLv2020] (2020). Discovery of a molecular glue promoting CDK12-DDB1 interaction to trigger cyclin K degradation. Elife.

[bMatthew2016] (2016). The importance of non-accessible crosslinks and solvent accessible surface distance in modeling proteins with restraints from crosslinking mass spectrometry. Mol Cell Proteomics.

[bOReilly2018] (2018). Cross-linking mass spectrometry: methods and applications in structural, molecular and systems biology. Nat Struct Mol Biol.

[bPerkins2010] (2010). Transient protein-protein interactions: structural, functional, and network properties. Structure.

[bQin2014] (2014). Weak protein complexes: challenging to study but essential for life. FEBS J.

[bReizer1992] (1992). Functional interactions between proteins of the phosphoenolpyruvate: sugar phosphotransferase systems of *Bacillus subtilis* and *Escherichia coli*. J Biol Chem.

[bSchilder2013] (2013). Formation of transient protein complexes. Curr Opin Struct Biol.

[bShi2015] (2015). A strategy for dissecting the architectures of native macromolecular assemblies. Nat Methods.

[bSmith1985] (1985). Filamentous fusion phage: novel expression vectors that display cloned antigens on the virion surface. Science.

[bSuh2007] (2007). Role of electrostatic interactions in transient encounter complexes in protein-protein association investigated by paramagnetic relaxation enhancement. J Am Chem Soc.

[bTang2020] (2020). Integrating non-NMR distance restraints to augment NMR depiction of protein structure and dynamics. J Mol Biol.

[bTang2006] (2006). Visualization of transient encounter complexes in protein-protein association. Nature.

[bVaynberg2006] (2006). Weak protein-protein interactions as probed by NMR spectroscopy. Trends Biotechnol.

[bWang2022] (2022). Characterization of protein unfolding by fast cross-linking mass spectrometry using di-ortho-phthalaldehyde cross-linkers. Nat Commun.

[bWheat2021] (2021). Protein interaction landscapes revealed by advanced *in vivo* cross-linking-mass spectrometry. Proc Natl Acad Sci USA.

[bWu2017] (2017). Atomic modeling of the ITS2 ribosome assembly subcomplex from cryo-EM together with mass spectrometry-identified protein-protein crosslinks. Protein Sci.

[bXing2014] (2014). Visualizing an ultra-weak protein-protein interaction in phosphorylation signaling. Angew Chem Int Ed Engl.

[bYang2018] (2018). Proximity-enhanced SuFEx chemical cross-linker for specific and multitargeting cross-linking mass spectrometry. Proc Natl Acad Sci USA.

[bYang2012] (2012). Identification of cross-linked peptides from complex samples. Nat Methods.

[bYu2018] (2018). Cross-linking mass spectrometry: an emerging technology for interactomics and structural biology. Anal Chem.

[bYu2020] (2020). Exploring spacer arm structures for designs of asymmetric sulfoxide-containing MS-cleavable cross-Linkers. Anal Chem.

[bZhao2019] (2019). A Pandas complex adapted for piRNA-guided transcriptional silencing and heterochromatin formation. Nat Cell Biol.

[bZhao2018] (2018). Structure and mechanogating mechanism of the Piezo1 channel. Nature.

[bZiemianowicz2019] (2019). Photo-cross-linking mass spectrometry and integrative modeling enables rapid screening of antigen interactions involving bacterial transferrin receptors. J Proteome Res.

