# Supplementary material for: Fast cross-linking by DOPA2 promotes the capturing of a stereospecific protein complex over nonspecific encounter complexes
Source: Biophys Rep. 2022 Dec 31;8(5-6):239–52. doi: 10.52601/bpr.2022.220014 (PMC10166511; doi:10.52601/bpr.2022.220014)
Supplement: Supplementary file 1 — Supplementary data to this article can be found online. [file br-8-239-S1.pdf]

Supplementary Materials

Table S1 The inter-molecular cross-links identified by DOPA2 or DSS in protein complex EIN/HPr. Cross-links were filtered by requiring FDR < 0.01 at the spectra level, E-value < 1×10^{-8} and spectral counts > 3. The estimated FDR at the residue pair level is zero

(**A**) The inter-molecular cross-links identified by DSS in the solution

Linked aa positions	Total Spec	Best E-value	Cα–Cα distance (Å) (PDB code: 3EZA)	Label
EIN(49)-HPr(49)	22	1.78×10^{-20}	26.48	In-solution
EIN(30)-HPr(49)	13	3.73×10^{-37}	29.90	In-solution
EIN(49)-HPr(72)	20	4.17×10^{-16}	35.22	In-solution
HPr(27)-EIN(30)	27	2.66×10^{-19}	35.30	In-solution
HPr(24)-EIN(30)	119	2.86×10^{-17}	36.78	In-solution
EIN(1)-HPr(49)	48	4.34×10^{-32}	44.96	In-solution
HPr(1)-EIN(30)	32	2.72×10^{-19}	46.92	In-solution
EIN(30)-HPr(72)	9	3.71×10^{-21}	47.21	In-solution
EIN(1)-HPr(40)	58	3.18×10^{-11}	48.40	In-solution
EIN(1)-HPr(24)	18	8.06×10^{-17}	49.07	In-solution
EIN(1)-HPr(27)	63	5.71×10^{-22}	53.15	In-solution
EIN(1)-HPr(72)	32	1.17×10^{-27}	60.88	In-solution
EIN(1)-HPr(1)	37	1.26×10^{-31}	64.70	In-solution

(**B**) As in **A**, but for cross-links identified by DOPA2

Linked aa positions	Total Spec	Best E-value	Cα–Cα distance (Å) (PDB code: 3EZA)	Label
HPr(49)-EIN(96)	5	1.92×10^{-19}	24.33	In-solution
EIN(49)-HPr(49)	21	1.05×10^{-17}	26.48	In-solution
EIN(30)-HPr(49)	83	3.73×10^{-31}	29.90	In-solution
HPr(24)-EIN(30)	47	1.95×10^{-12}	36.78	In-solution
HPr(24)-EIN(238)	11	5.31×10^{-16}	56.10	In-solution
HPr(27)-EIN(238)	6	1.66×10^{-20}	61.06	In-solution

(**C**) The inter-molecular cross-links identified by DSS in the gel

Linked aa positions	Total Spec	Best E-value	Cα–Cα distance (Å) (PDB code: 3EZA)	label
HPr(24)-EIN(58)	526	3.21×10^{-36}	15.38	In-gel
HPr(27)-EIN(58)	238	5.11×10^{-14}	19.62	In-gel
HPr(24)-EIN(49)	94	1.58×10^{-12}	22.32	In-gel
HPr(27)-EIN(49)	169	4.16×10^{-31}	23.42	In-gel
EIN(49)-HPr(49)	702	2.37×10^{-24}	26.48	In-gel
HPr(24)-EIN(175)	43	2.83×10^{-22}	34.05	In-gel
HPr(27)-EIN(30)	156	2.81×10^{-16}	35.30	In-gel
HPr(40)-EIN(49)	27	4.61×10^{-24}	36.39	In-gel
HPr(24)-EIN(30)	303	5.70×10^{-20}	36.78	In-gel
HPr(27)-EIN(29)	36	2.84×10^{-26}	36.85	In-gel
HPr(24)-EIN(29)	56	2.87×10^{-09}	37.81	In-gel
EIN(30)-HPr(40)	44	4.52×10^{-21}	38.01	In-gel
EIN(1)-HPr(40)	5	1.94×10^{-10}	48.40	In-gel
EIN(1)-HPr(24)	81	4.86×10^{-13}	49.07	In-gel
EIN(1)-HPr(27)	62	3.69×10^{-21}	53.15	In-gel

(**D**) As in **C**, but for cross-links identified by DOPA2

Linked aa positions	Total Spec	Best E-value	Cα–Cα distance (Å) (PDB code: 3EZA)	Label
HPr(24)-EIN(58)	233	8.11×10^{-40}	15.38	In-gel
HPr(27)-EIN(58)	504	4.98×10^{-23}	19.62	In-gel
HPr(24)-EIN(60)	15	1.31×10^{-09}	20.21	In-gel
HPr(27)-EIN(69)	45	1.65×10^{-14}	20.35	In-gel
HPr(49)-EIN(69)	28	1.58×10^{-18}	22.20	In-gel
HPr(24)-EIN(49)	127	2.89×10^{-33}	22.32	In-gel
HPr(27)-EIN(49)	86	3.44×10^{-23}	23.42	In-gel
HPr(49)-EIN(96)	638	3.05×10^{-41}	24.33	In-gel
EIN(49)-HPr(49)	78	6.50×10^{-18}	26.48	In-gel
EIN(30)-HPr(49)	623	1.18×10^{-34}	29.90	In-gel
EIN(29)-HPr(49)	24	6.25×10^{-16}	30.92	In-gel
HPr(40)-EIN(96)	56	1.95×10^{-10}	31.79	In-gel
HPr(27)-EIN(30)	274	2.13×10^{-21}	35.30	In-gel
HPr(40)-EIN(49)	77	3.25×10^{-28}	36.39	In-gel
HPr(24)-EIN(30)	209	8.37×10^{-23}	36.78	In-gel
HPr(27)-EIN(29)	78	3.22×10^{-26}	36.85	In-gel
HPr(24)-EIN(29)	37	1.21×10^{-10}	37.81	In-gel
EIN(30)-HPr(40)	130	2.27×10^{-31}	38.01	In-gel
EIN(29)-HPr(40)	24	5.62×10^{-16}	39.11	In-gel
HPr(1)-EIN(49)	66	6.96×10^{-26}	41.09	In-gel
HPr(1)-EIN(30)	7	2.05×10^{-19}	46.92	In-gel
HPr(24)-EIN(238)	229	2.10×10^{-13}	56.10	In-gel
HPr(49)-EIN(238)	65	1.76×10^{-20}	56.45	In-gel
HPr(40)-EIN(238)	4	3.15×10^{-09}	60.32	In-gel
HPr(27)-EIN(238)	205	2.49×10^{-32}	61.06	In-gel

Table S2 Inter-molecular cross-links between EIN and HPr identified from gel slices L1-L3

Cross-linker	Gel slices	Encounter complexes (ECs)		Stereospecific complex (SC)	
		# of X-links	# of spectra	# of X-links	# of spectra
DOPA2	L1	1	54	2	52
DOPA2	L2	8	308	5	146
DOPA2	L3	9	423	6	337
DSS	L1	1	17	3	30
DSS	L2	5	97	2	43
DSS	L3	6	278	5	150

The cross-links identification results were filtered by requiring FDR < 0.01 at the spectra level, E-value < 1×10^{-8} and spectral counts in each sample ≥ 2. The estimated FDR at the residue pair level is zero. The cross-links were classified according to their structural compatibility with either the stereospecific complex or the encounter complexes
